# Lymph node density as a surrogate marker for positive lymph nodes

**DOI:** 10.1038/sj.bjc.6606000

**Published:** 2010-11-23

**Authors:** T Van Gorp, A J Kruse, B F Slangen, R F Kruitwagen

**Affiliations:** 1 Division of Gynaecological Oncology, Department of Obstetrics and Gynaecology, MUMC+, GROW – School for Oncology and Developmental Biology, PO Box 5800, Maastricht 6202AZ, The Netherlands

**Sir,**

We read with interest the paper by Polterauer *et al* (2010) in which they evaluated the prognostic value of lymph node density (LND) in patients with lymph node-positive cervical cancer. LND is defined as the ratio of the number of metastatic lymph nodes to the total number of lymph nodes removed. They showed that LND>10% is associated with an impaired disease-free and overall survival. Lymph node involvement and lymph vascular space involvement have always been noted to be a poor prognostic factor in cancer of the cervix (Pecorelli, 2006), and thus this confirms the fact that the higher the number of lymph nodes involved, the worse the prognosis of the patient becomes. The benefit of using LND could be that it also incorporates the extent of surgical staging. Indeed, it is probably true that when you perform a complete lymph node dissection instead of a sampling you have more chance of finding all involved lymph nodes. However, there is more to it than positive lymph nodes and surgical skills.

Although the authors gave the mean/median number of removed lymph nodes and positive lymph nodes for all patients, we were not able to discern this information for the LND⩽10% or LND>10% group separately. We believe that this information is pivotal in a study that examines the ratio of both parameters. In a ratio, both the numerator and denominator have an equal role. The authors state that they performed a systemic lymphadenectomy in all patients. This means that the mean/median number of removed lymph nodes cannot be statistically different in both groups. As a consequence, only the denominator will become important, and thus the number of positive lymph nodes will become the prognostic factor. If for any reason the total number of lymph nodes removed in both groups is different, then one must question the reasons why. Did the surgeon stop the operation prematurely because during the operation he/she discovered bulky involved lymph nodes? This would make a complete systemic lymphadenectomy redundant, as the patient is already known to be lymph node positive based on these few lymph nodes. This is generally accepted as an indication for (chemo-)radiotherapy, and the combination of a radical surgery and pelvic (chemo-)radiotherapy will increase therapy-related morbidity (Quinn *et al*, 2006). Maybe the pathologist stopped looking intensively for other lymph nodes because he/she already found several positive lymph nodes.

Second, with respect to the technique used, they referred to a previous study in which a laparoscopic pelvic lymph node staging was described (Polterauer *et al*, 2008). In this study, patients were included between 1995 and 2007 and, remarkably, the median number of lymph nodes was 15, being more than 3 lymph nodes less than in the present study. Does this implicate a learning curve related to the laparoscopic technique used? If yes, once more a surgical bias is introduced.

Third, we question the cutoff value and how it was established. The authors mentioned that this was based on preliminary data from a study by Ooki *et al* (2007). We wonder how well oesophageal cancer can be compared with cervical cancer. Both organs are located in completely different anatomic regions, with a different lymph vessel drainage system and lymph node distribution. Did the authors try different cutoff points or was the 10% cutoff the only value examined? If the authors tried different cutoff points, one could argue that the authors should have used an independent validation set to validate this cutoff point.

Fourth, with a median of 18.5 lymph nodes (range 12–27), Polterauer *et al* demonstrated that they routinely performed complete lymph node dissections. As the ratio of the median number of involved lymph nodes (*n*=2) to the median number of removed lymph nodes (*n*=18.5) was 10.8%, removing one or two lymph nodes would more or less determine whether the patient belonged to the category of patients with LND⩽10% or LND >10%. This would implicate that the surgeon could influence to which prognostic group the patient will belong. After all, it suffices to remove more lymph nodes to shift the patient from the LND>10% group to the LND⩽10% group. It is contradictory that the surgeon has to remove more ‘healthy’ lymph nodes to ‘improve’ the prognosis of the patient. With more healthy lymph nodes the denominator will become bigger and thus the LND smaller. This strikes us as ironic.

In conclusion, as LND is influenced by surgical technique, anatomic circumstances and the quality and accuracy of the pathological analysis, we believe that LND is not an objective parameter and should not influence the decision on what kind of adjuvant treatment should be given to a patient. We believe that the number of positive lymph nodes is probably the true predictor, but as the number of patients in each of the different groups (1 *vs* 2 *vs* >2) was low, the authors were unable to reach significance. Using a ratio solved this problem by creating two arbitrary groups of patients and by using denominators that enlarged the difference between the numerators.
